# Measuring COVID-19 Related Health Literacy in Healthcare Professionals—Psychometric Evaluation of the HL-COV-HP Instrument

**DOI:** 10.3390/ijerph182211959

**Published:** 2021-11-14

**Authors:** Kati Hiltrop, Nina Hiebel, Franziska Geiser, Milena Kriegsmann-Rabe, Nikoloz Gambashidze, Eva Morawa, Yesim Erim, Kerstin Weidner, Christian Albus, Nicole Ernstmann

**Affiliations:** 1 Center for Health Communication and Health Services Research (CHSR), Department for Psychosomatic Medicine and Psychotherapy, University Hospital Bonn, 53127 Bonn, Germany; nicole.ernstmann@ukbonn.de; 2 Department for Psychosomatic Medicine and Psychotherapy, University Hospital Bonn, 53127 Bonn, Germany; nina.hiebel@ukbonn.de (N.H.); franziska.geiser@ukbonn.de (F.G.); milena.rabe@ukbonn.de (M.K.-R.); 3 Institute for Patient Safety, University Hospital Bonn, 53127 Bonn, Germany; nikoloz.gambashidze@ukbonn.de; 4 Department of Psychosomatic Medicine and Psychotherapy, University Hospital of Erlangen, Friedrich-Alexander University Erlangen-Nürnberg (FAU), 91054 Erlangen, Germany; Eva.Morawa@uk-erlangen.de (E.M.); Yesim.Erim@uk-erlangen.de (Y.E.); 5 Department for Psychotherapy and Psychosomatic Medicine, Carl Gustav Carus Faculty of Medicine, Technische Universität Dresden, 01307 Dresden, Germany; Kerstin.Weidner@uniklinikum-dresden.de; 6 Department of Psychosomatics and Psychotherapy, Medical Faculty and University Hospital Cologne, 50931 Cologne, Germany; christian.albus@uk-koeln.de

**Keywords:** health literacy, healthcare professionals, COVID-19, exploratory factor analysis, confirmatory factor analysis, SARS-CoV2-pandemic

## Abstract

Background: Thus far, there is no instrument available measuring COVID-19 related health literacy of healthcare professionals. Therefore, the aim of this study was to develop an instrument assessing COVID-19 related health literacy in healthcare professionals (HL-COV-HP) and evaluate its psychometric properties. Methods: An exploratory factor analysis, a confirmatory factor analysis, and descriptive analyses were conducted using data from *n* = 965 healthcare professionals. Health literacy related to COVID-19 was measured with 12 items, which were adapted from the validated HLS-EU-Q16 instrument measuring general health literacy. Results: Exploratory factor analysis demonstrated that 12 items loaded on one component. After removing one item due to its high standardized residual covariance, the confirmatory factor analysis of a one-factor model with 11 items showed satisfactory model fit (χ^2^ = 199.340, df = 41, χ^2^/df = 4.862, *p* < 0.001, RMSEA = 0.063, CFI = 0.963 and TLI = 0.951). The HL-COV-HP instrument showed good internal consistency (Cronbach’s alpha 0.87) and acceptable construct reliability. Conclusions: The HL-COV-HP is a reliable, valid, and feasible instrument to assess the COVID-19 related health literacy in healthcare professionals. It can be used in hospitals or other healt hcare settings to assess the motivation and ability of healthcare professionals to find, understand, evaluate, and use COVID-19 information.

## 1. Introduction

Individual health literacy can be defined as the knowledge, motivation, and ability of individuals to find, understand, evaluate, and use health information in the areas of health promotion, disease prevention, and healthcare in order to maintain or improve health and quality of life [1]. The question of how easily individuals find and understand health information and navigate in a health system depends on the complexity and the responsiveness of the environment [2]. The COVID-19 pandemic has increased peoples’ health information needs. People have to find, understand, trust, and use COVID-19 information to protect themselves and others [3]. They need COVID-19 related health literacy.

In a German population-based survey, the vast majority of respondents reported difficulties dealing with COVID-19 information, especially difficulties judging whether they could trust media information on COVID-19 [4]. These difficulties are related to general health literacy: surveys reveal disparities in COVID-19 related knowledge, attitudes, and behaviors according to people’s health literacy and language [4,5]. The underestimation of poor health literacy in the general population as a serious public health problem is getting even more relevant in pandemic times [6]. Especially critical health literacy, understood as individuals’ ability to reflect on complex health issues and critically assess the information available, is needed during the COVID-19 pandemic to promote, enhance and encourage adequate decisions and health behavior [7].

A higher level of health literacy, especially e-health literacy, is associated with COVID-19 awareness [8], positive attitudes towards preventive strategies against COVID-19 [9,10,11,12,13,14], and a higher adherence to prevention guidelines [8,15]. Health literacy is positively associated with COVID-19 vaccination acceptance [16] and can mitigate the negative effects of healthcare system distrust on vaccination willingness [17,18]. Consequently, indicators of low health literacy are associated with higher COVID-19 infection rates [19]. Health literacy is also a protective factor for mental health during the COVID-19 pandemic [12,20,21].

While there is a growing body of research on aspects of COVID-19 related health literacy of the general population [4,15,16,22] or specific populations, e.g., migrants, children, students, or patients [8,9,10,11,13,19,20,21,23], little is known about the COVID-19 related health literacy of healthcare professionals, such as physicians, nurses, or psychologists. Since 2020, many studies have investigated the knowledge, attitude, and behavior of healthcare professionals worldwide [24,25,26,27,28,29,30,31,32,33,34]. The results indicate a lack of adequate knowledge about COVID-19 in many cases (6–42%) [24,25,27,32]. Healthcare professionals having a higher education were found to have better knowledge about COVID-19 [28,33,35]. Strong significant correlations were found between knowledge, attitude, and behavior [26,34]. However, data on the COVID-19 related health literacy that allow the analysis of healthcare professionals’ knowledge, motivation, and ability to find, understand, evaluate, and use the information on COVID-19 is still missing. Such data are needed to identify subdimensions associated with lower literacy levels to develop tailored interventions that enable healthcare professionals to protect themselves and their patients [35]. A German study indicates that less vaccination knowledge and more vaccination hesitancy of healthcare professionals is associated with information-seeking behavior in messenger services or online video platforms rather than using scientific sources [36]. Comprehensive and longitudinal surveys on such associations are needed.

According to a recent scoping review, the development and validation of instruments that measure pandemic-related health literacy are needed [37]. Thus far, there is no instrument available measuring COVID-19 related health literacy of healthcare professionals. Therefore, it is the aim of this study to develop an instrument assessing COVID-19 related health literacy in healthcare professionals (HL-COV-HP) and evaluate its psychometric properties. We decided to adapt a self-assessment instrument since validated short forms exist.

## 2. Materials and Methods

### 2.1. Study Design and Data Collection

The online survey data were collected from April to July 2020 as part of the VOICE study [38,39] on stress and resilience in the COVID-19-pandemic, in cooperation with an ongoing research project on resilience in religion and spirituality. Participants were included in the study when they were >18 years old, worked in the healthcare sector, had a residence/working place in Germany, had sufficient German language skills, and gave informed consent. To recruit healthcare professionals, the link to the online survey was distributed Germany-wide via professional associations, advertisements in intranet websites of hospitals, contacting CEOs of hospitals (primarily in North-Rhine Westphalia), and newsletters to hospital staff. The 15-min online survey consisted of a basic set of questions related to the resilience of healthcare professionals and an additional set, which included among others the HL-COV-HP items. In total, *n* = 1232 healthcare professionals participated, of which *n* = 986 answered the additional part of the survey. The study was approved by the Ethics Committee of the Medical Faculty of the Rheinische Friedrich Wilhelm University Bonn (reference number: 125_20).

### 2.2. Measure

To measure health literacy related to COVID-19 in healthcare professionals, the HL-COV-HP instrument was developed. The items of the HL-COV-HP were adjusted to the COVID-19 pandemic and the healthcare setting by a team of health service researchers. The items were adapted from the HLS-EU-Q16 instrument, which was a validated short version of the HLS-EU-Q47 [40]. Both instruments measured general individual health literacy based on the respondents’ ease with finding, understanding, evaluating, and using the information in terms of the domains healthcare, disease prevention, and health promotion. The items of both HLS-EU-Q16 and the HL-COV-HP use a scale from “very easy,” “fairly easy,” “fairly difficult,” to “very difficult” [40]. The items scored from 1 to 4, with higher sum scores indicating lower health literacy [40]. While the long-version had a multidimensional structure, the short form HLS-EU-Q16 had primarily psychometric properties of a unidimensional scale [41]. The HL-COV-HP items were tested in 2 cognitive think-aloud interviews with healthcare professionals.

### 2.3. Analysis

Missing data per item was 2.1%, and cases with missing data were deleted listwise, leading to *n* = 965 cases that were included in the analyses. All analyses were carried out with SPSS v25 (descriptive analyses, exploratory factor analysis, Cronbach’s alpha) and AMOS v27 (confirmatory factor analysis).

#### 2.3.1. Descriptive Measures

The descriptive analyses comprised the mean, standard deviation, median, skewness, minimum, maximum, discrimination, and item difficulty for each item. Item discrimination (corrected item-total-correlation) indicates if the single items correlate with the score from the total set of items and should be at least 0.3 [42]. Item difficulty indicates on a scale from 0–100 how difficult the item was for the respondents in the sample, with lower values indicating higher difficulty.

#### 2.3.2. Exploratory Factor Analysis

To investigate the factor structure of the set of items, exploratory factor analysis (EFA) was applied. To evaluate the suitability of each item and the set of items for EFA, the measure of sampling adequacy (MSA; >0.5) and the Kaiser–Meyer–Olkin measure (KMO; >0.5 mediocre, >0.7 good, >0.8 great, >0.9 superb) were used [42]. A significant Bartlett’s test would indicate that correlations between items were significantly different from zero, and data were appropriate for EFA [42]. Factors were extracted with principal component analysis [42]. The amount of extracted factors was guided by the Kaiser criterion (eigenvalues > 1) and the scree plot [42]. A minimum of 3 items should load on one factor [43]. Factor loadings > 0.4 would be considered significant and cross-loadings < 0.4 acceptable [42,43]. The percentage of nonredundant residuals with absolute values > 0.05 should be less than 50%, although no strict rules exist [42].

#### 2.3.3. Confirmatory Factor Analysis

To estimate how well the data fit the original unidimensional model, a confirmatory factor analysis (CFA) was carried out. The model fit was measured using the following criteria and thresholds: normed χ^2^ (χ^2^/df ≤ 2 good, ≤5 acceptable), root mean square error of approximation (RMSEA < 0.07), comparative fit index (CFI ≥ 0.95), and Tucker–Lewis index/non-normed fit index (TLI ≥ 0.95) [43].

#### 2.3.4. Internal Consistency and Convergent Validity

Construct reliability (CR > 0.6 acceptable, >0.7 good) and the average extracted variance (AVE ≥ 0.5) were used as indicators for convergent validity [43]. Cronbach’s alpha was calculated as an indicator of internal consistency of the instrument and would be considered good if >0.8 [42].

## 3. Results

### 3.1. Sample Characteristics and Descriptive Results

Of *n* = 965 healthcare professionals, 23.0% were physicians and 28.4% nurses. Moreover, 15.1% were spiritual care workers, 9.1% were medical-technical staff, and 2.2% were psychologists. Other occupations (17.3%) included among others scientific staff, study nurses, or physiotherapists. Participants were mainly female (72.2%), and many age groups were represented in the sample. Table 1 shows the sample characteristics.

The set of 12 items and their descriptive statistics are presented in Table 2. Measured on a scale from 1 = very easy to 4 = very difficult, the means per item varied between 1.38 and 2.43 with median values from 1 to 2. The corrected item-total correlations ranged from 0.464 to 0.688 and item difficulty from 12.8 to 47.4.

### 3.2. Exploratory Factor Analysis

Principal component analysis with Varimax rotation was carried out on the 12 items. Sampling adequacy was confirmed with the Kaiser–Meyer–Olkin measure (KMO = 0.90; superb), and MSA values for the individual items were at least 0.810, therefore, exceeding the minimum threshold of 0.5 [42]. A highly significant Bartlett’s test of sphericity χ^2^ (66) = 4817.210, *p* < 0.001, indicated the appropriateness of data to conduct EFA for these data [42]. Two components had eigenvalues over Kaiser’s criterion of 1 and, in combination, explained 54.65% of the variance. The scree plot justified retaining either one or two components. Theoretic assumptions, additionally considering the fact that the Kaiser’s criterion can tend to overestimate the number of factors [42], lead to retaining one component explaining 44.69% of the variance. A total of 68% of non-redundant residuals with absolute values greater than 0.05 occurred. The final results of the component analysis with 12 items loading on one component are presented in Table 3.

### 3.3. Confirmatory Factor Analysis

**Figure 1:**
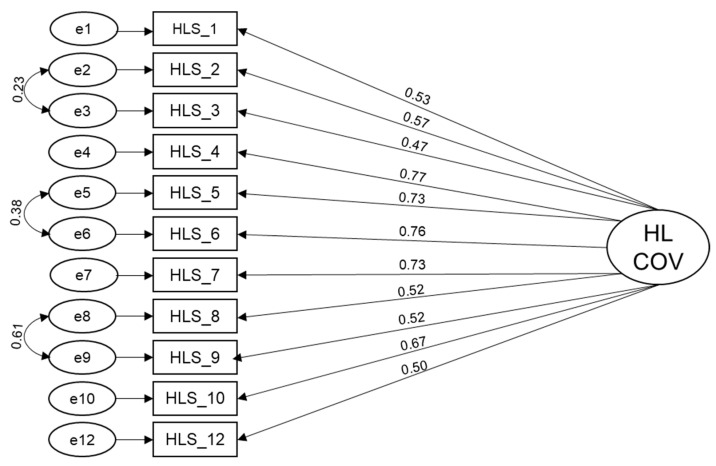
Confirmatory model of the HL-COV-HP instrument (*n* = 965).

The confirmatory factor analysis replicated the one-factor structure of the original instrument. Item 11 (“to decide based on information from the media how you can protect yourself against COVID-19?”) was removed due to its high standardized residual covariance. After allowing three error terms to correlate, the following model fit measures can be reported for the final model: χ^2^ = 199.340, df = 41, χ^2^/df = 4.862, *p* < 0.001, RMSEA = 0.063, CFI = 0.963, and TLI = 0.951. Figure 1 shows the confirmatory model of the HL-COV-HP. Cronbach’s alpha for the 11 items was 0.87, CR 0.905, and AVE 0.447.

## 4. Discussion

It was the aim of this study to evaluate the psychometric properties of the German version of an instrument assessing COVID-19 related health literacy in healthcare professionals (HL-COV-HP) based on an online survey with physicians, nurses, medical-technical staff, psychologists, spiritual workers, and other occupational groups being in direct contact with patients suffering from COVID-19.

Acceptance of the HL-COV-HP items among participants was good. Item difficulties usually ranged from easy to medium. When applying the HL-COV-HP instrument, attention should be paid to items one and four as they showed higher item difficulty (<20). If the item difficulty remains high in other samples, the item wordings might need to be adjusted or items removed. Overall, our analyses showed that the HL-COV-HP has satisfactory psychometric properties. The exploratory factor analysis revealed a one-component solution explaining 44.69% of the total variance. Although a solution should account for at least 60% of the total variance, it is not uncommon to accept solutions accounting for less variance as satisfactory in social sciences [43]. About 68% of the non-redundant residuals had absolute values greater than 0.05. Ideally, a maximum of 50% of the non-redundant residuals exceed 0.05, but no strict rules exist [42]. Cronbach’s alpha suggests good internal consistency. In terms of convergent validity, the construct reliability exceeded the desired threshold, while the AVE failed to reach 0.5. According to Fornell and Larcker [44], convergent validity is adequate if the AVE was >0.4 and composite reliability >0.6. The confirmatory factor analysis confirmed the unidimensional structure. One item was dropped due to its high standardized residual covariances. High standardized residual covariances indicate differences between the observed covariances and the estimated covariances based on the model, therefore, the smaller the standardized residuals, the better is the model fit [43]. Items associated with several large standardized residuals are most likely dropped [43]. The three correlated error terms in the final model represent common modifications in factor analysis because they allow to statistically consider correlations of items of the instrument [45]. The overall fit indices χ^2^/df, RMSEA, CFI, and TLI demonstrate a good fit and thus underline the unidimensional structure. These findings are in line with analyses on the structure of the HLS-EU-Q16 instrument [41], which was the foundation for the items of the HL-COV-HP.

The descriptive results show COVID-19 related health literacy deficits, especially in the domains of evaluating and using COVID-19 information for their own safety. These results are in line with prior research of general health literacy based on the HLS-EU-Q16 [46]. These first results for the group of healthcare professionals are worth considering since healthcare professionals are used to dealing with health information and have access to reliable sources, scientific evidence, and research results. However, they report difficulties even in using the information provided by their own organizations for their own safety. In interpreting our results, we have to consider the time of our survey at the beginning of the pandemic. Future studies will have to verify these findings. Still, the general health literacy and thus the COVID-19 related health literacy of healthcare professionals are important dimensions of health literate healthcare organizations [47,48,49,50]. By preparing the workforce for the COVID-19 pandemic and by enhancing the health literacy skills of the staff, healthcare organizations can protect and support both the health of their employees and the safety of their patients. The HL-COV-HP will allow the analysis of healthcare professionals’ knowledge, motivation, and ability to find, understand, evaluate, and use the information on COVID-19 to identify subdimensions associated with lower literacy levels in specific subgroups of healthcare professionals (e.g., in terms of age, profession, years of professional experience). Thus, tailored interventions that enable healthcare professionals to protect themselves and their patients can be developed.

### Strengths and Limitations

We developed the HL-COV-HP instrument based on a comprehensive theoretical framework [1] and based on one of the associated validated and widely used questionnaires assessing the individual health literacy, the HLS-EU-Q16 [41]. In comparison to existing instruments (e.g., [4]), the HL-COV-HP is shorter and specifically tailored to measure COVID-19 related health literacy in healthcare professionals. The analysis was based on a large sample of various occupational groups working with COVID-19 patients. We performed both exploratory and confirmatory factor analysis. However, there are limitations to consider in interpreting the results. The psychometric evaluation was based on cross-sectional data. We were not able to examine the test-retest-reliability of the HL-COV-HP instrument. Further validation should include longitudinal data analysis to test the responsiveness and sensitivity to change. Thus far, an instrument assessing aspects of COVID-19 related health literacy in healthcare professionals was missing. Therefore, we were not able to evaluate the criterion validity by comparison with an existing gold standard. Our results were obtained from professionals working in German hospitals and thus may reflect COVID-19 related health literacy specifically for professionals in this national healthcare system. The sample might overrepresent spiritual care workers due to the fact that they were specifically addressed as an occupational group with patient contact during the COVID-19 pandemic. Moreover, female participants could be overrepresented despite the fact that some of the occupations in our sample are more often carried out by females in Germany (e.g., nurses).

## 5. Conclusions

The HL-COV-HP is a reliable, valid, and feasible instrument to assess the COVID-19 related health literacy in healthcare professionals. It can be used in hospitals or other healthcare settings to assess the motivation and ability of healthcare professionals to find, understand, evaluate, and use COVID-19 information. It may be used to examine differences between subgroups of professionals, e.g., with or without contact to COVID-19 patients, with more or less years of professional experience, or between different professions. Once the responsiveness and sensitivity to change of the instrument will be tested, it can be used to monitor changes of COVID-19 related health literacy or to examine the effectiveness of interventions in pre-/post- study designs. The HL-COV-HP could be used in different healthcare settings, such as the outpatient setting. Since COVID-19 developments vary between countries, the HL-COV-HP can be used in different healthcare systems. Moreover, the use in other (virus-caused) pandemic situations could be possible.

## Figures and Tables

**Table 1:** Sample characteristics (*n* = 965 healthcare professionals).

	Frequency (*n*)	Percentage (%)
Sex		
Male	266	27.6
Female	697	72.2
Diverse	2	0.2
Age		
18−30 years	162	16.8
31−40 years	199	20.6
41−50 years	191	19.8
51−60 years	324	33.6
>60 years	89	9.2
Occupation		
Physician	222	23.0
Nurse	274	28.4
Medical-technical staff	88	9.1
Psychologist	21	2.2
Spiritual care worker	146	15.1
Employees in administration in direct contact with patients	47	4.9
Other	167	17.3
Previous infection with SARS-CoV2		
Yes	14	1.5
No	488	50.6
I do not know	463	48.0

**Table 2:** HL-COV-HP items.

	Item	M	SD	Md	S	Min	Max	r_i_	P_i_
	How easy/difficult is it for you…								
1.	to find information about COVID-19?	1.38	0.545	1.00	1.109	1	4	0.477	12.8
2.	to find out where to get professional help if you have COVID-19 yourself?	1.62	0.702	2.00	0.890	1	4	0.538	20.8
3.	to find information on behaviors that are good for your mental wellbeing during the COVID-19 pandemic?	2.07	0.818	2.00	0.385	1	4	0.464	35.6
4.	to understand information on how to protect yourself against COVID-19?	1.46	0.623	1.00	1.232	1	4	0.675	15.3
5.	to understand information about possible treatment for COVID-19?	1.77	0.775	2.00	0.778	1	4	0.668	25.6
6.	to understand Information about the risk factors associated with severe COVID-19?	1.73	0.780	2.00	0.835	1	4	0.688	24.2
7.	to assess which of your everyday habits increase the risk of suffering from COVID-19 yourself?	1.60	0.690	1.00	0.974	1	4	0.642	20.1
8.	to assess whether the information about COVID-19 in the media can be trusted?	2.43	0.859	2.00	0.012	1	4	0.582	47.7
9.	to assess whether information about COVID-19 from scientific sources is reliable?	2.38	0.821	2.00	0.033	1	4	0.578	46.1
10.	to use the information available to you to decide how to behave in the event of being infected with COVID-19 yourself?	1.83	0.727	2.00	0.562	1	4	0.643	27.7
11.	to decide based on information from the media how you can protect yourself against COVID-19?	2.05	0.818	2.00	0.500	1	4	0.573	35.0
12.	to decide based on information from your employer how you can protect yourself against COVID-19?	2.01	0.874	2.00	0.585	1	4	0.490	33.7

Notes: Scale: 1 = very easy, 2 = fairly easy, 3 = fairly difficult, 4 = very difficult; M: Mean, SD: Standard deviation, Md: Median, S: Skewness, Min: Minimum, Max: Maximum, r_i_ = Discrimination (corrected item-total-correlation), P_i_ = Difficulty, *n* = 965.

**Table 3:** Exploratory model of the HL-COV-HP instrument (*n* = 965).

	Item	Factor Loading
1.	Find information about COVID-19	0.563
2.	Find out where to receive professional help when falling ill with COVID-19	0.620
3.	Find information on behaviors which are good for the psychological well-being during the COVID-19 pandemic	0.543
4.	Understand information about how to protect myself from COVID-19	0.758
5.	Understand information about a potential treatment of COVD-19	0.757
6.	Understand information about risk factors for a severe course of COVID-19	0.774
7.	Assess which daily routines increase the risk to fall ill with COVID-19	0.730
8.	Assess whether information about COVID-19 is reliable on the media	0.649
9.	Assess whether information about COVID-19 is reliable from scientific sources	0.644
10.	Decide based on present information how to behave when falling ill with COVID-19	0.718
11.	Decide based on information from media how to protect myself from COVID-19	0.644
12.	Decide based on information from employer how to protect myself from COVID-19	0.567
	% of explained variance	44.69

## Data Availability

Please contact Franziska Geiser (franziska.geiser@ukbonn.de) for questions concerning data availability.
